# Effects of house dust mite subcutaneous immunotherapy in real-life. Immunological and clinical biomarkers and economic impact analysis

**DOI:** 10.1016/j.waojou.2023.100789

**Published:** 2023-06-15

**Authors:** Cristiano Caruso, Stefania Colantuono, Barbara Tolusso, Clara Di Mario, Giovanni Fancello, Marilena La Sorda, Giorgio Celi, Mario Caringi, Anna Volterrani, Desideria Descalzi, Elisa Gremese, Maurizio Sanguinetti, Antonio Gasbarrini, Giorgio Walter Canonica

**Affiliations:** a Department of Medical and Surgical Sciences, Fondazione Policlinico Universitario A. Gemelli, IRCCS, Università Cattolica del Sacro Cuore, Rome, Italy; b Immunology Research Core Facility, Fondazione Policlinico Universitario A. Gemelli, IRCCS, Rome, Italy; c Division of Clinical Immunology, Fondazione Policlinico Universitario A. Gemelli, IRCCS, Rome, Italy; d Dipartimento Scienze di Laboratorio e Infettivologiche, Fondazione Policlinico Universitario A. Gemelli, IRCCS, Rome, Italy; e ASST Carlo Poma, Mantova, Italy; f Sapienza University of Rome, Italy; g Personalized Medicine, Asthma and Allergy, Humanitas Research Hospital, Rozzano, MI, Italy

**Keywords:** Subcutaneous immunotherapy, Basophil, Biomarkers, Economic impact analysis, Real-life

## Abstract

**Background:**

Etiology of allergic rhinitis and asthma is frequently associated with house dust mite sensitization and allergen immunotherapy (AIT) represents the only disease modifying treatment. In a real world setting, clinicians would benefit from biomarkers to monitor or predict response to AIT.

**Methods:**

Twenty-four consecutive house dust mite (HDM) mono-sensitized rhinitic patients, treated with subcutaneous immunotherapy (SCIT) as per clinical practice, were enrolled. Multiple *in vitro* biomarkers such as basophil activation (BAT), IL-10 levels, and molecular allergen-specific IgE were performed during HDM SCIT, to monitor the effects of AIT and then correlated to *in vivo* scores (VAS, CMSS, RQLQ). Nasal cytology was performed at baseline and after 6 and 12 months of treatment. Finally, the economic impact of SCIT in this cohort of patients was evaluated.

**Results:**

Clinical biomarkers confirmed to be useful to monitor AIT efficacy. As for laboratory biomarkers, BAT showed a reduction trend, particularly for D2C1, suggesting that this is a useful parameter in monitoring patients. IL-10 levels tend to remain stable or slightly decrease during treatment. The economic analysis confirmed the favorable impact of immunotherapy.

**Conclusions:**

In this cohort of patients, SCIT confirmed its effectiveness in reducing symptoms and drug utilization. Clinical scores confirmed to be valid in monitoring patients and their response. BAT demonstrated to be useful in monitoring more than predicting response. Further studies are needed to better explore the usefulness of these biomarkers in AIT.

To the Editor,

In real-life an allergist needs outcome measures of allergen immunotherapy (AIT) that may indicate certain decision points (ie, change of extract, extension of treatment) and assist on patients’ adherence.^1^ Nonetheless, despite our knowledge of the mechanisms of AIT, no defined biomarker predictive of response has been identified so far. In a recent real-life study, patients with allergic conditions had a mean prevalence of sensitization to house dust mite (HDM) of 21.7%.^2^ The recommended methods for treating HDM allergy include avoidance of allergens, symptomatic medications, and AIT. Symptoms of allergic rhinitis (AR) can be reduced by symptomatic treatment using antihistamines, topical or inhalant corticosteroids (ICS) and bronchodilators when asthma is associated with this condition. AIT is effective as a long-term option and, above all, is the only disease-modifying option.^3^ Double-blind placebo-controlled clinical studies proved efficacy of HDM allergoid in terms of symptom medication score (CMSS).^4^

The poor long-term adherence is known to affect the efficacy of AIT. In the case of injection AIT (SCIT), one of the main determinants is the inconvenience for patients to undergo prolonged build-up phases. Thus, simplifying the time schedule of the induction protocol could be effective in increasing the adherence to SCIT.^5^ Also costs of treatment affect compliance to specific SCIT, demonstrating that the direct and indirect costs of SCIT are the most important factors for treatment discontinuation. However, on the other hand, numerous evidences demonstrated the cost-effectiveness of AIT.^6^

The aim of this study was to evaluate potential clinical and nonclinical monitor/response biomarkers for AIT. Multiple *in vitro* biomarkers such as basophil activation (BAT), IL-10 levels, and molecular allergen specific IgE were performed during HDM SCIT, to monitor the effects of AIT and then correlated to *in vivo* scores (VAS, CMSS, RQLQ). Finally, we evaluated the economic impact of SCIT in this cohort of patients.

## Methods

Consecutive HDM mono-sensitized rhinitic patients, eligible for AIT as per clinical practice, were included in the study between January and July 2019 and evaluated for a 12-month follow-up period. The presence of well-controlled asthma associated with rhinitis did not represent exclusion criteria for the study. The diagnosis and treatment of AR and asthma were appropriate, according to current guidelines (the Allergic Rhinitis and its Impact on Asthma, ARIA, and Global Initiative for Asthma, GINA).^7^^,^^8^ All patients, tested for a defined panel of allergens,^9^ were diagnosed by either positive skin prick tests or positive serum specific IgE (sIgE) to HDMs (≥0.35 kUA/L). The allergen extract used in skin prick testing for HDM was manufactured by Hal Allergy (Leiden, Holland). Patients' demographic data (age, gender, medical history), allergic disease diagnosis and duration, SCIT composition and date of initiation, and SCIT administration schedule were registered and evaluated. SCIT indications were based on EAACI guidelines.^10^ All patients were treated with the same HDM allergoid as the most frequently prescribed SCIT product in Italy, which has already been tested in clinical trials (Purethal, Hal Allergy).^11^ All patients or their parents or legal guardians provided signed informed consent. The study was approved by the Ethics Committee in 2019 (ID number 2371). Patients were clinically evaluated at 3 timepoints (baseline, 6, and 12 months). BAT was used to assess the expression of basophil activation markers after antigen stimulation, aiming to investigate the changes in basophil activation in response to the inhalant allergens of HDM (*Dermatophagoides pteronyssinus* and *Dermatophagoides farinae*) during immunotherapy. Serum IL-10 levels were determined using commercially High Sensitivity ELISA kit (Quantikine® HS human IL-10 R&D – BioTechne, UK), according to the manufacturer's protocol. BAT procedure and IL-10 serum level determination are more deeply described in Supplementary material section.

ALEX© test (MacroArrayDX -Wien, Austria) was performed in a small proportion of patients, in order to explore the sensitization to infrequent molecular components and monitor them during treatment.^12^ Nasal cytology was performed at baseline and after 6 and 12 months of AIT using a pencil-shaped disposable nasal curette with a small distal cup (Rhinoprobe®, Nasal scraping®). May-Grünwald-Giemsa (MGG) staining was used to identify inflammatory nasal cells and optical microscopy for reading, using a semiquantitative grading.^13^ For each patient, concomitant medications were revised, and comorbidity symptom score (CMSS), rhinosinusitis quality of life questionnaire (RQLQ), and visual analog scape (VAS) questionnaires (further details available in Supplementary material) were performed, in order to analyze any modification of symptoms during therapy and consequently any change in concomitant medications. Patients who achieved improvement of all clinical outcome were defined *complete responders* and patients who achieved improvement in at least 2 out of 3 items were considered *partial responders*, after one-year treatment. Statistical analysis is described in detail in the Supplementary material section. Finally, an economic analysis attempt was performed, assessing the direct costs of disease (ie, the reduction in anti-reactive concomitant therapy vs the costs of AIT itself) to determine the cost-saving effect of AIT, since AIT and the majority of these medications are not reimbursable in our country and are fully charged to the patient.

## Results

Twenty-four HDM mono-sensitized rhinitic patients (9 females and 15 males) were included in the study. The demographic and clinical characteristics of the entire cohort of patients at baseline are illustrated in Table 1. The median age was 22 years and the median basal values of symptom score were 70 ± 28,3, 2 ± 1,31 and 2,3 ± 1,34 for VAS, CMSS and RQLQ, respectively. All patients were administered regularly according to the product schedule and remained adherent to therapy for at least 1 year.

**Table 1:** Demographic and clinical characteristics of patients.

F/M	9/15
Age (median, years)	22
Disease duration (median, years)	10
VAS (median ± SD)	70 ± 28,3
CMSS (median ± SD)	2 ± 1,31
RQLQ (median ± SD)	2,3 ± 1,34

Abbreviations: CMSS: combined medication and symptoms score; RQLQ: rhinoconjunctivitis quality of life questionnaire; SD: standard deviation; VAS: visual analogue scale

**Fig. 1:**
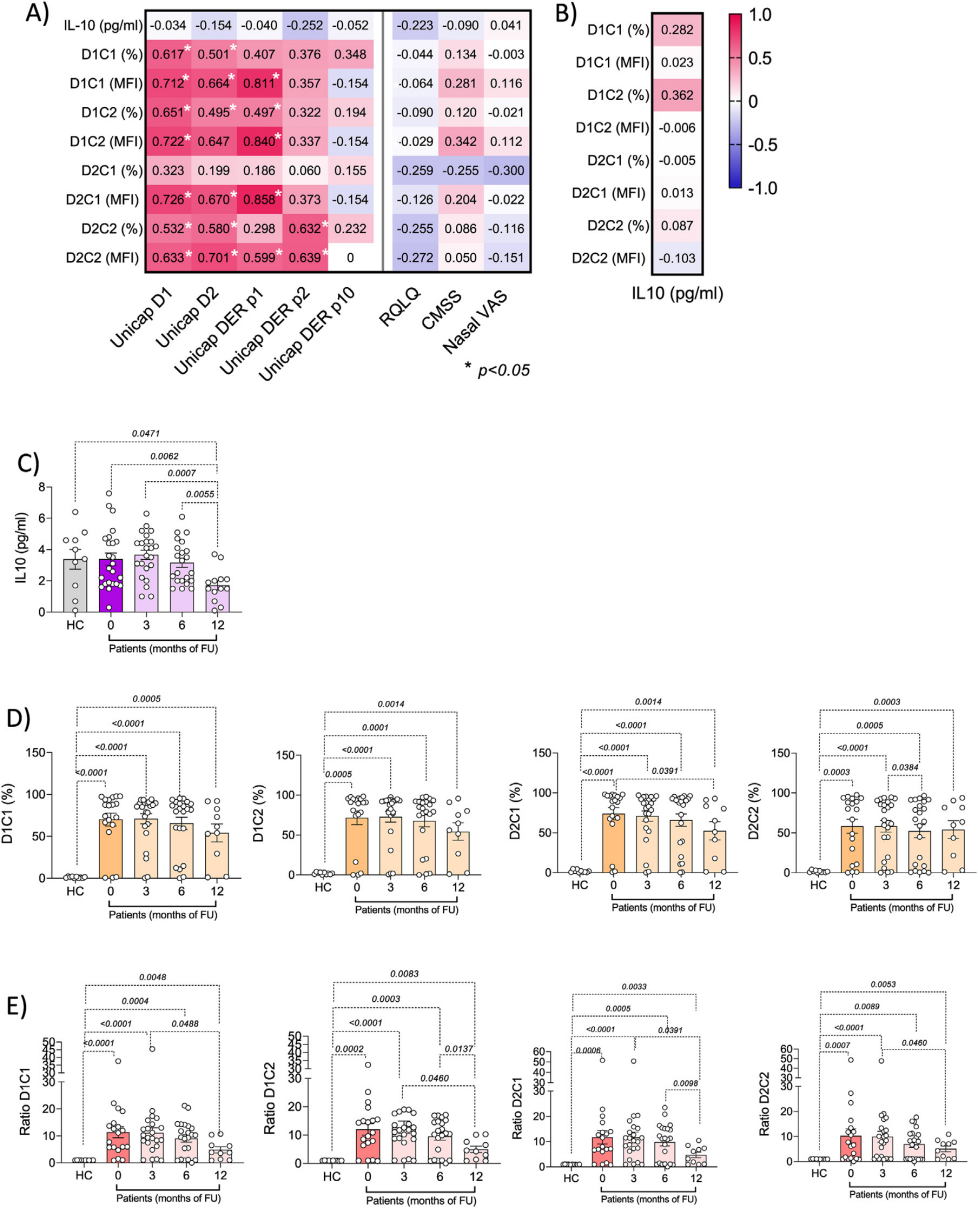
A) Spearman's rank correlation between specific IgE levels (i.e. D1, D2, DERp1, DER p2 and DERp10) or clinical data (RQLQ, CMSS or Nasal VAS) and BAT (using D1C1/D1C2 or D2C1/D2C2) or IL-10 plasma levels at baseline in HDM mono-sensitized rhinitic patients. Numbers represent R^2^ and the stars represent a *p* value < 0.05. B) Spearman's rank correlation between IL-10 plasma levels at baseline and BAT (using D1C1/D1C2 or D2C1/D2C2). C) IL-10 plasma levels in healthy controls (HC) and in patients at baseline and during treatment (3, 6 and 12 months). D) Flow cytometric analysis to evaluate the percentage of basophils activated with D1C1, D1C2, D2C1 and D2C2 in healthy controls (HC) and in patients during treatment (Mann-Whitney test). E) Flow cytometric analysis to evaluate the ratio of basophils activated with D1C1, D1C2, D2C1 and D2C2 in healthy controls (HC) and in patients during AIT (Mann-Whitney test)

Fig. 1A shows the correlation between baseline parameters of disease activity and laboratory assays. BAT and IL-10 plasma levels demonstrated no utility in characterizing disease activity in mono-sensitized patients. As expected, BAT percentages using the two antigens (ie, D1 and D2) at the 2 different concentrations (ie, C1 and C2) were significantly higher in patients than in healthy controls. Similarly, the percentage of activation did not differ during treatment among patients (Fig. 1D), except for D2C1 percentage that was significantly lower at 12 months when compared to baseline (Fig. 1E).

**Fig. 2:**
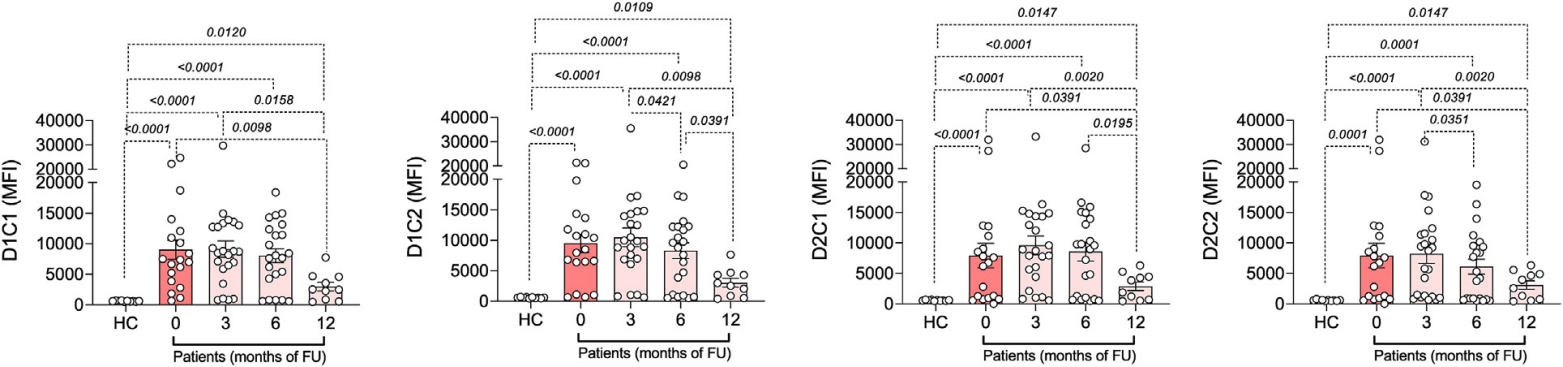
Flow cytometric analysis to evaluate the Mean of fluorescence intensity (MFI) of D1C1, D1C2, D2C1 and D2C2 activated basophils in healthy controls (HC) and in HDM mono-sensitized rhinitic patients at baseline and during AIT (3, 6 and 12 months)

Indeed, if we consider the ratio between the positive and the negative MFI value for every BAT analysis, we found a reduction trend that becomes significant at 12 months of follow-up (Fig. 2), particularly for D2C1, suggesting that this could be a useful parameter in monitoring patients.

Unfortunately, BAT did not demonstrate useful in predicting response, in fact no significant differences in percentage of activation and ratio were found between responders and non-responders/partial responders (Supplemental figure 1).

Nonetheless, when considering the whole group of patients, having D2C1 ratio values ≥ 6.37 at baseline (cut-off value from ROC analysis) (Supplementary Figure 2) identifies patients more likely to achieve VAS-defined response after 12 months (91.7%) compared to patients with baseline D2C1 ratio values < 6.37 [50.0%, p = 0.05)]

No correlations were found between BAT at baseline and IL-10 plasma levels (Fig. 1B). Moreover, differently from other evidences, IL-10 levels tend to remain stable or slightly decrease during treatment (Fig. 1C).

Assessment of specific IgE by ImmunoCap and ALEX© test, when performed, did not show significant variation during therapy, confirming that these tools are not suitable as monitor biomarkers (data not shown).

CMSS and VAS questionnaires demonstrated to be valid tools in monitoring nasal symptom changes and drug utilization. CMSS reduced to 1,1 ± 0.94 and 1 ± 0.93 and VAS score to 45 ± 26 and 30 ± 26 at 3 and 6 months, respectively. RQLQ reduced to 1.2 ± 1 and 0,94 ± 1,2 at 3 and 6 months respectively. One-year scores didn't show any additional clinical improvement.

Nasal cytology showed a reduction in the mean percentage of the eosinophil count from baseline (3.35 cells) to 6 month (2.25 cells) and 12-month follow-up (1.12 cells). Although eosinophils consisted of the majority of inflammatory cells in the nasal secretion of most AR patients, neutrophils were found to be increased in some patients, 15% of patients in the current study.

## Discussion

The fear of being infected with COVID-19 has been the most common reason for SCIT drop-out during the COVID-19 pandemic. Nonetheless delays in SCIT administration can deteriorate clinical symptoms.^14^ As for adherence, 100% of our patients treated with the standard schedule completed the first-year treatment, with a very high adherence rate. However, due to the COVID-19 pandemic outbreak, some patients did not perform one-year evaluation at site, were administered by family doctor and were asked to complete questionnaires remotely. Only patients who performed a clinical on-site evaluation underwent blood and serum samples for BAT and IL-10 at 12 months follow-up. From this point of view, as mentioned above, it is possible that the duration of the follow-up partially affected some results.

BAT did not demonstrate useful neither to better characterize patients at baseline nor to predict response to immunotherapy, but the reduction trend in MFI ratio at 12 months follow-up suggested a certain usefulness in monitoring patients during therapy. As for IL-10, the trend during therapy could be counter-intuitive, but it may be partially due to the limited number of patients who achieved a 12-month laboratory follow-up.

Multiplex test like ALEX© has increased the recognition of different sensitization patterns (ie, Der p 21, Der p 23) and arise some interesting issues. One patient showed a single sensitization to Der p 23, nonetheless clinical symptoms scores improved during treatment and observation. Another patient showed a multiple sensitization to different allergens (Der f1, Der f2, Der p1, Der p2, Der p23, Der p7) and specific IgE showed a marked increase especially from T0 to T6. However, from a clinical point of view, the patient experienced a good relief of symptoms at the beginning of the observation, even if not persistent at the end of the follow-up.

At the end of observation only 2 non responder patients were reported. One of them showed a stable value of VAS and CMSS, but reported an improvement in RQLQ. The other one underwent ENT surgery because of trauma some months after starting therapy, so the response to the questionnaires could be biased and were not considered for evaluation.

It is also to consider that the forced lockdown heavily exposed patients to indoor allergens such as HDM, so the clinical improvement observed in the others at the end of follow-up is absolutely remarkable.

### Economic impact analysis

Overall, studies examining the pharmaco-economics have demonstrated the cost-effectiveness of AIT.^15^ With respect to clinical beneﬁt, economic evaluation on immunotherapy, as compared with pharmacotherapy, indicated that the cost-effectiveness of immunotherapy primarily depends on the duration of the clinical beneﬁt following treatment cessation. Di Bona et al demonstrated that the cost-effectiveness of SCIT was sensitive to changes in rhino-conjunctivitis total symptom scores (RTSS) and consequently to changes in Quality-adjusted life-years (QALY) estimates.^6^ When direct and indirect costs were considered, the per patient annual costs were significantly less from SCIT than standard therapy. Furthermore, none of these medications have a disease-modifying effect. A German study compared the cost-effectiveness of SCIT in addition to standard therapy versus the use of SCIT alone and showed that the increasing treatment rates is a cost-effective strategy with an incremental cost-effectiveness ratio.^16^

At the end of the study, an estimate of the economic impact was attempted in this cohort of patients, but only direct costs (ie, the current costs of SCIT itself and those of symptomatic anti-reactive therapy) were considered in this case. Given to the limited follow-up, no considerations about long term benefits of immunotherapy, such as the effects on asthma prevention and the onset of new allergen sensitizations, were possible and also the indirect costs (ie, work or school-day loss/saving) have been excluded from the evaluation, to avoid the COVID-19 pandemic bias, during which most patients stayed at home anyway because of lockdown.

All patients included in the study belonged to Italian regions where no refunds for SCIT is provided to patients. Moreover, in our country all local therapy and some antihistamines (more recent and selective molecules) are charged to patient. Given to the perennial sensitization to HDM, as per clinical practice, a 10-months anti-reactive therapy is usually needed by patients and has been taken into account for costs evaluation.

In our study the combination of SCIT with standard therapy confirmed a direct cost-savings of 140 € per patient during the first year of therapy but, for the reasons mentioned above, it is plausible to think that the cost effectiveness will be incremental during years.

## Conclusion

In this cohort of patients, clinical biomarkers confirmed to be useful to monitor AIT efficacy. As for laboratory biomarkers, BAT showed a reduction trend, particularly for D2C1, suggesting that it could be relevant for patient monitoring and may help physicians in making therapeutic decisions. Probably the sample size and the short period of observation are factors to consider for other biological biomarkers. Real-life studies with long-term outcomes are therefore necessary for definitive considerations.

## Abbreviations

AIT, allergen immunotherapy: SCIT, subcutaneous immunotherapy: BAT, basophil activation test: HDM, house dust mite.

## Acknowledgments

Thank you to Dr. S. De Federicis and Dr. R. Mariotti (Hal Allergy, Italia) for the collaboration in conceptualizing the idea of the study and their continuous support and to *Fondazione Roma* (Via del Corso 239, 00187, Rome) for the relevant and continuous support to CEMAD in clinical research and its applications.

## Funding

No funding was received for the manuscript.

## Availability of data and materials

Data and materials are available upon request.

## Authors’ contributions

CC and SC conceptualized the study, drafted the article and reviewed it; GC, AV and MC contributed in acquisition and interpretation of data; BT, CDM, MLS, GF and DDS contributed in analysis, interpretation of data and statistical analysis; EG, GWC, MS and AG reviewed the manuscript critically for important intellectual content; all author reviewed the final version of the manuscript, gave the final approval of the version to be published and agrees to be accountable for all aspects of the work related to its accuracy or integrity.

All authors agree with the final version of the manuscript and its publication.

## Ethics approval

The study was approved by the Ethics Committee of the Fondazione Policlinico Universitario A. Gemelli IRCCS in 2019 (ID number 2371).

## Declaration of competing interest

The authors declare no conflict of interest for this manuscript.

## Disclosure

The authors report no conflicts of interest in this work.

## Appendix A Supplementary data

Supplemental figure 1 . A) Percentage of basophils activated with D1C1, D1C2, D2C1 and D2C2 in HDM mono-sensitized rhinitic patients, responders (R_light blue) and partial or no responders (PR/NR_light red) to AIT compared to healthy controls (HC). B) Ratio of basophils activated with D1C1, D1C2, D2C1 and D2C2 in HDM mono-sensitized rhinitic patients, responders (R) and partial or no responders (PR/NR) to AIT compared to healthy controls (HC). Supplemental figure 2. Receiver operating characteristic (ROC) analysis. Capacity of D2C1 ratio values at baseline more likely to achieve VAS-defined response under treatment within 12 months in patients with HDM rhinitis. The area under the curve (AUC) was 0.872 ± 0.102 (Sensitivity: 69,2%, Specificity: 100,0%; *p* = *0.05*. The cut-off value of D2C1 ratio at baseline was 6,37. Supplemental figure 1
