# Supplementary material for: Effects of house dust mite subcutaneous immunotherapy in real-life. Immunological and clinical biomarkers and economic impact analysis
Source: World Allergy Organ J. 2023 Jun 15;16(6):100789. doi: 10.1016/j.waojou.2023.100789 (PMC10362513; doi:10.1016/j.waojou.2023.100789)
Supplement: Supplemental figure 1 — . A) Percentage of basophils activated with D1C1, D1C2, D2C1 and D2C2 in HDM mono-sensitized rhinitic patients, responders (R_light blue) and partial or no responders (PR/NR_light red) to AIT compared to healthy controls (HC). B) Ratio of basophils activated with D1C1, D1C2, D2C1 and D2C2 in HDM mono-sensitized rhinitic patients, responders (R) and partial or no responders (PR/NR) to AIT compared to healthy controls (HC). Supplemental figure 2. Receiver operating characteristic (ROC) analysis. Capacity of D2C1 ratio values at baseline more likely to achieve VAS-defined response under treatment within 12 months in patients with HDM rhinitis. The area under the curve (AUC) was 0.872 ± 0.102 (Sensitivity: 69,2%, Specificity: 100,0%; p = 0.05. The cut-off value of D2C1 ratio at baseline was 6,37. [file mmc1.docx]

**Supplementary material**

The BAT was performed using the Flow CAST kit (Bühlmann Laboratories AG, Schönenbuch, CH) according to the manufacturer’s instructions to detect the expression of the basophil activation surface marker CD63 in peripheral blood. Allergens for the BAT (Dermatophagoides pteronyssinus [D1], Dermatophagoides farinae [D2]) were purchased from Bühlmann Laboratories (Bühlmann Laboratories AG, Schönenbuch, CH). Two concentrations for each allergen were tested [225 ng/mL (C1) and 45 ng/mL (C2) for BAG-D1 and 22,5 ng/mL (C1) and 4,5 ng/mL (C2) for BAG-D2] and monitored at different time point (T0, T3, T6 and T12) for each patient. For positive controls, both a monoclonal anti-FcεRI antibody and N-formyl-methionyl-leucyl-phenylalanine (fMLP; 2 μM) were used. For the negative background control, 50 μL of stimulation buffer was used. Briefly, 100 μL of stimulation buffer, 50 μL of allergen (or controls) and 20 μL of staining reagent (containing anti-CD63-FITC and anti-CCR3-PE monoclonal antibodies) were added to 50 μL of EDTA whole blood. Then the tubes were incubated at 37°C for 15 minutes, and stimulation was stopped by adding 2 mL of lysis buffer. After centrifugation for 5 minutes at 500 × g, the supernatant was decanted and 300 μL of washing buffer were added to each tube. The cells were resuspended by gently vortexing before flow cytometry analysis. Flow cytometry analysis was performed using a BD FACSCanto (BD Biosciences, San Jose, CA, USA), and the data were analyzed using BD Diva-software (BD Biosciences). Upregulation of the activation marker CD63 was calculated as the percentage of CD63-positive cells compared with the total number of basophils; results were also expressed as median fluorescence intensity (MFI). To select the basophil population we set a gate on CCR3 positive cells with low side scatter SSC (CCR3pos / SSClow), to descart the eosinophil cells also positive for anti-CCR3.

IL-10 *.* Blood samples were collected at baseline and after 6 and 12 months of AIT; sera were stored at -80 °C until analysis. Each IL-10 assessment was run in duplicate for each sample. The OD value at 450 nm was measured. The assay range was 0,8-50 pg/mL and the sensitivity was 0.17 pg/mL.

Statistical analysis was performed using SPSS V. 20.0 (SPSS. Chicago, Illinois, USA) and Prism software (GraphPad-9, San Diego, California, USA). Categorical and quantitative variables were described as frequencies, percentage and mean ± SEM. Data on demographic and clinical features were compared between patients by the non-parametric Mann-Whitney U test or χ2 test, as appropriate. Spearman’s rank correlation test was used for correlation in all analyses. A receiver operating characteristics (ROC) curve analysis of BAT analysis (D2C2) at baseline related to VAS achievement after at least 12 months was performed to obtain relevant thresholds allowing the prediction of BAT at baseline. The non-parametric ROC plot uses all the data, makes no parametric assumption and provides unbiased estimates of sensitivity and specificity. The optimal cut-off point was determined to yield the maximum corresponding sensitivity and specificity. For all the analyses, a p<0.05 was considered as statistically significant and all tests were 2-tailed, unless otherwise indicated.
